# Diagnostic Accuracy of Inflammatory Biomarkers in Differentiating Acute Appendicitis From Other Acute Abdomen and Predicting Disease Severity: A Prospective Comparative Cross‐Sectional Study

**DOI:** 10.1002/hsr2.72562

**Published:** 2026-05-25

**Authors:** Sintayehu Admas, Bisrat Birke Teketelew, Nigusie Alemu, Berhanu Woldu, Lyusira Marelgn, Getu Girmay, Arega Zenaw, Abebe Birhanu, Yemataw Gelaw

**Affiliations:** ^1^ Department of Hematology and Immunohematology, School of Biomedical and Laboratory Sciences, College of Medicine and Health Sciences University of Gondar Gondar Ethiopia; ^2^ Department of Medical Laboratory Sciences, College of Medicine and Health Sciences Mizan Tepi University Mizan Aman Ethiopia; ^3^ Department of Immunology and Molecular Biology, School of Biomedical and Laboratory Sciences, College of Medicine and Health Sciences University of Gondar Gondar Ethiopia; ^4^ Department of Clinical Chemistry, School of Biomedical and Laboratory Sciences, College of Medicine and Health Sciences University of Gondar Gondar Ethiopia; ^5^ Department of Medical Microbiology, School of Biomedical and Laboratory Sciences, College of Medicine and Health Sciences University of Gondar Gondar Ethiopia

**Keywords:** acute appendicitis, diagnostic accuracy, inflammatory biomarkers

## Abstract

**Introduction:**

Acute appendicitis (AA) is one of the most common clinical conditions for emergency surgery. The diagnosis of acute appendicitis remains challenging for surgeons. Researchers are still looking for a parameter that is less expensive and easily available for diagnosis. The main aim of the study was to determine the diagnostic accuracy of inflammatory biomarkers for AA and the severity of inflammation.

**Methods:**

A prospective comparative cross‐sectional study was conducted among 161 participants (80 with acute appendicitis and 81 non‐AA abdominal pain controls) recruited using a consecutive sampling technique. The study was conducted from August 21, 2024 to January 16, 2025. In this study, we have investigated the diagnostic utility of white blood cells (WBC), neutrophil to lymphocyte ratio (NLR), systemic inflammatory index (SII) and systemic inflammation response index (SIRI) for AA. Normality was assessed using the Shapiro–Wilk test and visual inspections. Mean differences for WBC were compared using the Independent sample *t*‐test, while the Mann–Whitney U test was employed for others. ROC curve analysis was done for statistically significant parameters (*p* value < 0.05 with 95%CI). Post‐hoc power analysis was performed using G*Power 3.1.9.7 to ensure study robustness.

**Result:**

Males account for 57.76% of participants. The mean age of non‐AA and AA group was 26.47 ± 8.53 and 24.34 ± 9.77 years, respectively. The AA group demonstrated significantly higher WBC, NLR, SII and SIRI levels compared to non‐AA. Significantly higher WBC, NLR, SII and SIRI levels were observed in complicated AA (CAA) groups relative to simple AA (SAA) groups. The area under the ROC curve (AUC) of WBC, NLR, SII, and SIRI to diagnose AA was 0.814, 0.834, 0.816, and 0.814, respectively. WBC, NLR, SII, and SIRI can predict CAA with AUC of 0.722, 0.781, 0.770, and 0.715, respectively.

**Conclusion:**

Inflammatory markers, including WBC, NLR, SII, and SIRI have acceptable diagnostic performance for AA. Additionally, WBC, NLR, SII and SIRI possess potential for differentiating CAA from SAA. It may be better for surgeons to consider the level of NLR, SII and SIRI for the diagnosis of AA and to determine its severity. However, further studies on a larger sample size are recommended to validate their clinical utility.

**Abbreviations**
AA: acute appendicitis
AUC: area under the curve
CAA: complicated acute appendicitis
CBC: complete blood count
CRP: C‐reactive protein
ED: emergency department
NA: negative appendectomy
NPV: negative predictive value
RLQ: right lower quadrant
ROC: receiver operating characteristics
SAA: simple acute appendicitis
SE: sensitivity
SII: systemic immune‐inflammation index
SIRI: systemic inflammation response index
SP: specificity
UOGCSH: University of Gondar comprehensive specialized hospital
US: ultrasound
WBC: white blood cell

## 1 Introduction

Acute appendicitis (AA) is an inflammation of the vermiform appendix, and it is one of the most common acute abdominal pain indicated for emergency surgery globally [1]. It is characterized by an acute onset of pain at the right iliac fossa associated with anorexia, fever, nausea, and vomiting with marked tenderness at the right iliac fossa [2]. AA is classified into simple (SAA) and complicated (CAA) forms according to the extent of inflammatory involvement [3]. The CAA is associated with perforation, appendiceal mass, abscess formation, and peritonitis [4].

While delayed or missed diagnosis escalates complication rates and associated morbidity and mortality, premature surgical intervention unnecessarily increases the frequency of negative appendectomies [5, 6]. Imaging modalities have high accuracy to diagnose AA, though there are still challenges regarding resource accessibility, especially in rural areas of developing countries, including our country, Ethiopia [7]. Clinical decision‐making in AA traditionally relies on standardized scoring systems, such as the Alvarado score, the Appendicitis Inflammatory Response (AIR) score, and the Adult Appendicitis Score (AAS). While these tools integrate clinical symptoms with basic laboratory findings like total WBC count and C‐reactive protein (CRP), their diagnostic accuracy remains inconsistent [8]. Specifically, these scores often fail to accurately differentiate between SAA and CAA [9]. As a result, AA remains the subject of intense study for researchers hoping to develop a diagnostic marker that is reliable, inexpensive, noninvasive, and easily accessible [10, 11].

Various Complete blood count (CBC) parameters have emerged as useful diagnostic biomarkers of many inflammatory diseases [12, 13, 14, 15]. However, a single blood routine parameter is not ideal for evaluating the prognosis of patients with inflammatory diseases. Recently, the combined ratios of CBC parameters are used as indices of inflammation and are helpful in the diagnosis of many diseases. The most common are the neutrophil‐to‐lymphocyte ratio (NLR), the platelet‐to‐lymphocyte ratio (PLR), and the monocyte‐to‐lymphocyte ratio (MLR) [16, 17, 18].

More recently, several emerging multi‐parameter indices have been proposed to better capture the complexity of systemic immune activation. Systemic immune‐inflammation index (SII) is a simple, accessible, and inexpensive index reflecting the balance between a patient's immune status and inflammation level [19]. SII simultaneously reflects both pro‐inflammatory activity (neutrophilia and thrombocytosis) and stress‐induced immune suppression (lymphopenia), making it more acceptable as an inflammatory indicator compared to individual markers like WBC count or CRP [20]. Its prognostic and diagnostic roles are reported in various disorders, including cardiovascular disease, pre‐eclampsia, and cancers [21, 22, 23, 24].

Recently, the diagnostic utility of the SII in appendicitis has been validated by a systematic review and meta‐analysis conducted by Arredondo Montero et al. [25], which demonstrated high pooled sensitivity and specificity across multiple cohorts. The investigators suggested that SII emerges as a useful index for diagnosing AA and differentiating CAA and SAA. BIDIAP (Biomarkers for the Diagnosis of Appendicitis in Pediatrics) index, a newly developed diagnostic score by Arredondo Montero et al. [26], enhances the detection of AA by integrating the SII with established clinical and laboratory parameters, demonstrating the clinical benefit of combining systemic inflammatory indices with traditional diagnostic metrics. Another CBC‐based innovative inflammatory biomarker is the systemic inflammation response index (SIRI). SIRI has been used as an inflammatory marker in many diseases, including COVID‐19 [27] and AA [27, 28]. It is a marker reflecting the balance between immune response and inflammation, offering prognostic information in inflammatory conditions [29].

The SII and SIRI offer a more comprehensive and integrative assessment of systemic inflammatory status than individual biomarkers, as these composite indices balance regulatory elements (lymphocytes) against pro‐inflammatory components (neutrophils, monocytes, and platelets) [30]. The clinical utility of these markers extends beyond acute infections to the complex microenvironments of neoplastic diseases. For instance, Troise et al. [31] demonstrated that these biomarkers provide critical diagnostic support in detecting occult neck metastases in early‐stage oral squamous cell carcinoma (OSCC). Similarly, Abbate et al. [32] highlighted that inflammatory biomarkers, particularly SIRI, serve as valuable adjuncts to cytological analysis in the differential diagnosis of salivary gland tumors. These findings underscore the versatility of SII and SIRI as sensitive indicators of systemic stress across a diverse spectrum of pathological conditions.

Although these inflammatory biomarkers have been studied for their potential value in the diagnosis of AA [28, 33, 34, 35, 36, 37], there is still a significant degree of inconsistency and discrepancies regarding their accuracy. These inconsistencies may stem from inconsistent determination of cutoff values, variations in study populations, including geographical or genetic differences. This gap highlights the need for region‐specific research to validate the applicability of these tools in our healthcare context. By emphasizing our prospective design and consecutive sampling in a specific demographic, we have more clearly defined how this study aims to resolve these discrepancies.

In resource‐limited settings where advanced imaging, such as computed tomography (CT), is frequently inaccessible or delayed, evaluating the utility of cost‐effective biomarkers is of significant clinical importance. Given the lack of prior regional data, our findings suggest that these indices could serve as accessible adjuncts to local triage algorithms, facilitating surgical prioritization. Consequently, this study aimed to evaluate the diagnostic potential of the neutrophil to lymphocyte ratio (NLR), SII, and SIRI for identifying AA and assessing disease severity at the University of Gondar Comprehensive Specialized Hospital (UoGCSH) and Gondar Ayira General Hospital, Northwest Ethiopia.

## 2 Methods and Materials

### 2.1 Study Design, Period, and Setting

This was a prospective comparative cross‐sectional study conducted from August 21, 2024 to January 16, 2025. The study was conducted in Northwest Ethiopia, at the University of Gondar Comprehensive Specialized Hospital and Gondar Ayira General Hospital. UOGCSH is one of the oldest teaching hospitals in Ethiopia. Gondar Ayira General Hospital is a general hospital found in Azezo woreda, Gondar, serving the community since 2023. Diagnosis of AA in both hospitals is based on clinical, laboratory, and imaging (abdominal ultrasound) examinations.

### 2.2 Ethical Approval and Consent to Participate

Ethical approval was obtained from the ethical committee of the School of Biomedical and Laboratory Sciences, University of Gondar, Gondar, Ethiopia (Ref. No: SBMLS/812/2024)*.* All study participants provided written informed consent, and for those study participants under 18 years of age, written informed consent and assent were also obtained from the parents/guardians. A support letter was obtained from UOGCSH and Gondar Ayira General Hospital Medical Directors. The confidentiality of the study participants was maintained since a unique code was assigned to each study participant. Participants had the right to refuse to participate in the study at any time. The study was conducted in accordance with the Helsinki Declaration for Biomedical Research [38].

### 2.3 Source and Study Population

The source population of the current study was all those with abdominal pain who were suspected of AA coming to the emergency department. On the other hand, the study population was all patients who underwent appendectomy with a diagnosis of AA (the cases) and those with non‐AA abdominal pain after US examination as a comparative group. The comparison group consisted of patients who presented with acute abdominal pain but were ultimately diagnosed with non‐inflammatory conditions (renal colic, simple ovarian cysts, and intussusception) based on a combination of clinical evaluation and imaging (ultrasonography) that ruled out AA and other inflammatory diseases. Because abdominal ultrasound has variable sensitivity, particularly in early AA, these patients were monitored clinically until discharge to ensure no symptoms of appendicitis developed. Thus, the probability of inadvertently including false‐negative appendicitis cases is substantially reduced.

### 2.4 Eligibility Criteria

#### 2.4.1 Inclusion Criteria

The study enrolled symptomatic patients presenting with acute abdominal pain suggestive of appendicitis. Cases were defined as participants with a clinical and ultrasonographic (US) diagnosis of AA who subsequently underwent appendectomy. For the assessment of disease severity (SAA vs. CAA), inclusion was limited to cases confirmed as AA‐positive by histopathology. The comparative group consisted of symptomatic patients presenting with acute abdominal pain who were confirmed to be negative for AA. This was established through an initial negative abdominal US examination followed by continuous clinical monitoring until hospital discharge.

### 2.5 Exclusion Criteria

Children under 5 years of age were excluded. In this age group, the immune‐inflammatory response is characterized by extreme systemic reactivity and rapid pathological progression toward perforation, which may not be captured by diagnostic thresholds established for older cohorts. Conversely, children aged 5 years and above and adults are more likely to present with classic clinical symptoms and a more “staged” inflammatory response [39]. Patients with known chronic inflammatory disorders, hypertension, diabetes mellitus, asthma, kidney disease, heart failure, or established hematological disorders were excluded. Moreover, the study didn't include those patients who had received anticoagulants, antibiotics, or anti‐inflammatory medications and pregnant women. For the severity sub‐analysis, participants initially suspected of AA but found to have a normal appendix (NA) upon histopathological examination were excluded.

### 2.6 Sample Size and Sampling Technique

The sample size for this study was determined using the diagnostic index formula for continuous variables [40], assuming a 95% confidence level and a statistical power of 80%. We utilized an estimated Area Under the Curve (AUC) of 0.93 for the NLR as a benchmark, derived from a previous study [41]. This allowed to detect a significant diagnostic performance for the composite inflammatory markers under investigation. By taking 10% of the non‐response rate, the total sample size was found to be 161 (80 AA and 81 non‐AA abdominal pain participants).

A consecutive sampling technique was used to recruit study participants.

### 2.7 Study Variables

The study involved dependent (WBC, NLR, SII, and SIRI) and independent variables (sociodemographic characteristics (i.e., sex and age) and clinical characteristics (right lower quadrant (RLQ) tenderness, anorexia, nausea, vomiting, fever, migratory pain, rebound tenderness, diameter of the appendix, and severity of AA)).

### 2.8 Data Collection

Sociodemographic and clinical characteristics of the participants were collected using a pretested structured questionnaire through face‐to‐face interviews (Supporting Information S1: Table S1). The data were collected under the supervision of the principal investigator from those providing a written consent to participate in the study. The initial clinical evaluation of all participants was conducted using the standardized parameters of the Alvarado scoring system, including the assessment of pain migration, anorexia, nausea/vomiting, RLQ tenderness, rebound tenderness, fever, and leukocytosis. However, the final surgical decision was not based on the cumulative score alone; instead, it integrated these clinical findings with laboratory data and US. Ultimately, the definitive diagnosis of AA was confirmed via the “gold‐standard” histopathological examination of the surgically resected specimens.

### 2.9 Laboratory Tests

#### 2.9.1 Blood Collection and Complete Blood Count Analysis

Venous blood was collected by a professional laboratory technologist using the syringe method from the study participants at admission to the ED. The blood was added to an Ethylene Diamine Tetra Acetic acid (K_3_EDTA) tube (Qianxinan, Guizhou, China) for CBC analysis. Then, the collected blood was analyzed using an automated 5‐part hematology analyzer, Mindray BC‐5150 (Shenzhen, P.R. China), to determine CBC parameters. The machine uses an electrical impedance principle to count PLT and RBC. An electrical impedance principle is based on the fact that electrical resistance will occur when blood cells in an electrical conductor reagent solution pass through the small detection aperture. For WBC 5‐part differential analysis and WBC counting, the analyzer uses the light scatter principle in addition to impedance. In light scattering, forward scatter is related to cell size, while side scatter is related to cell complexity (granularity). Mindray BC‐5150 5‐part hematology analyzer displays about 25 parameters. NLR, SII, and SIRI were calculated from the given CBC parameters. NLR is the ratio of absolute neutrophil count to absolute lymphocyte count [42]. SIRI was calculated as absolute neutrophil count * absolute monocyte count/absolute lymphocyte count, whereas SII was obtained as absolute neutrophil count * platelet count/absolute lymphocyte count [35].

### 2.10 Histopathology Procedures and Examination

After an open appendectomy was done, the removed specimen was immediately fixed with 10% formalin and sent to the histopathology laboratory. Then, the pathologists examined the gross appearance of the specimen and performed gross sectioning (at the tip, middle, and proximal). The specimen after gross sectioning underwent an overnight processing. After overnight processing, the tissue underwent paraffin embedding, sections were then stained with Hematoxylin and Eosin (H/E) stain. Finally, the tissue was mounted with DPX and examined microscopically by pathologists.

### 2.11 Operational Definitions

Complicated acute appendicitis—perforated or gangrenous appendicitis or the presence of periappendicular abscess (mass) or peritonitis defined by histopathology [4, 43].

Negative appendectomy—removal of a normal appendix in the absence of an inflammatory process confirmed by histopathology [44].

Simple acute appendicitis—acutely inflamed appendix, phlegmonous/suppurative in the absence of abdominal mass, gangrene, perforation, or abscess around the appendix confirmed by histopathology [43].

The diagnostic performance of each parameter was defined as perfect, excellent, good, fair, poor (weak), and failed if AUC = 1, 0.9 ≤ AUC < 1, 0.8 ≤ AUC < 0.9, 0.7 ≤ AUC < 0.8, 0.6 ≤ AUC < 0.7, and 0.5 ≤ AUC < 0.6, respectively [5, 45].

### 2.12 Data Processing and Statistical Analysis

Data were entered into Epi Data version 4.7 and exported to SPSS version 26 (SPSS Inc., Chicago, USA) for analysis. Categorical variables were summarized using frequencies and percentages. The distribution for continuous inflammatory biomarkers (WBC, NLR, PLR, SII, and SIRI) was evaluated using the Shapiro–Wilk test and visual inspection of histograms and Q–Q plots. For variables following a normal distribution, specifically WBC count in the comparison between SAA and CAA with *p* > 0.05, the independent samples *t*‐test was employed to compare mean differences, with results expressed as mean ± SD. Conversely, all other biomarkers exhibited a non‐normal distribution (*p* < 0.05). Consequently, the Mann–Whitney U test was utilized for group comparisons, and these parameters are presented as median (interquartile range, IQR). Moreover, post‐hoc power analysis was conducted using GPower 3.1.9.7. For parameters showing statistically significant mean/median difference (*p* value < 0.05 with 95% CI), receiver operating characteristics (ROC) curve analysis was performed to determine the AUC, sensitivity (SE), and specificity (SP) of the parameters. The best cut‐off value for each parameter was determined using the Youden index (J), which is calculated as J = maximum (SE + SP − 1), that is, by taking the cut‐off value corresponding to the maximal value of J [45]. The positive predictive value (PPV) and negative predictive value (NPV) of each parameter were determined using a 2*2 cross tabulation.

## 3 Result

### 3.1 Socio‐Demographic Characteristics of Study Participants

A total of 161 study participants (129 from UOGCSH and 32 from Gondar Ayera General Hospital) were enrolled in this study. There were 81 non‐AA and 80 AA participants. The majority of the participants in both groups were males (44 (54.32%) and 49 (61.25%) in no‐AA and AA, respectively). The mean age of non‐AA and AA was 26.47 ± 8.53 and 24.34 ± 9.77 years, respectively. No statistically significant difference was observed in terms of sex and age between the two groups (*p* > 0.05) (Table 1).

**Table 1:** Socio‐demographic characteristics of study participants.

Characteristics		non‐AA, *N* (%)	AA, *N* (%)	*p* value
Sex	Male	44 (54.32)	49 (61.25)	0.373
	Female	37 (45.68)	31 (38.75)	
Age	(Mean ± SD)	26.47 ± 8.53	24.34 ± 9.77	0.142
	Range	6–50	5–53	

Abbreviation: AA, acute appendicitis.

### 3.2 Clinical Characteristics of Acute Appendicitis Groups

Direct and rebound RLQ tenderness, respectively, was observed in 79 (98.75%) and 44 (55.00%) of AA groups. Moreover, leukocytosis (WBC > 10*10^3^/µL) was seen in 53 (66.25%) of the case group (Table 2).

**Table 2:** Clinical characteristics of AA groups.

Characteristics	AA, *N* (%)	Non‐AA, *N* (%)	*p* value
Migratory pain			0.000
Yes	58 (72.50)	27 (33.3)	
No	22 (27.50)	54 (66.7)	
RLQ tenderness			0.000
Yes	79 (98.75)	64 (79.0)	
No	1 (1.25)	17 (21.0)	
Rebound tenderness			0.003
Yes	44 (55.00)	26 (67.9)	
No	36 (45.00)	55 (32.1)	
Fever (T° > 37.5°C)			0.237
Yes	42 (52.50)	44 (54.3)	
No	38 (47.50)	37 (45.7)	
Anorexia			0.893
Yes	58 (72.50)	60 (74.1)	
No	22 (27.50)	21 (25.9)	
Nausea and/or vomiting			0.595
Yes	64 (80.00)	62 (76.5)	
No	16 (20.00%)	19 (23.5)	
Leukocytosis (WBC > 10^3^/µL)			0.000
Yes	53 (66.25)	31 (38.3)	
No	27 (33.75)	50 (61.7)	

Abbreviations: RLQ, right lower quadrant; WBC, white blood cells.

**Figure 1:**
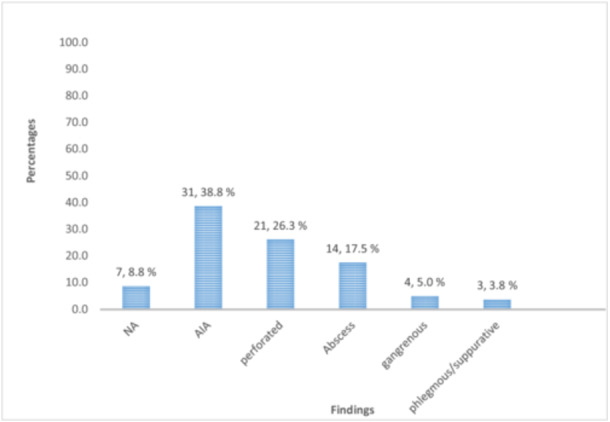
Histopathology findings of the AA groups (*n*, percentage) (AIA, acutely inflamed appendicitis; NA, negative appendectomy).

Based on histopathology findings, 39 (48.75%) of the AA group were found to be grouped under CAA (21 perforated, 14 abscess, and four gangrenous). SAA was observed in 34 (42.5%) of the AA group (31 acutely inflamed appendicitis (AIA) and three phlegmonous/suppurative appendicitis). The remaining seven (8.75%) participants underwent NA (Figure 1).

### 3.3 Comparative Analysis of Inflammatory Biomarkers Between Study Groups

In this study, the median difference of both SII and SIRI between AA and non‐AA were statistically significant (*p* < 0.001). Statistically significant median difference was also observed regarding WBC count and NLR (*p* < 0.05) between the two groups (Table 3).

**Table 3:** CBC parameters analysis of the study groups.

Parameters	Median (IQR)		*p* value
	Non‐AA (*N* = 81)	AA (*N* = 80)	
WBC^a^	7.15 (6.09–10.10)^b^	12.71 (9.02–15.93)^b^	< 0.001*
NLR^a^	3.38 (2.45–6.17)	9.39 (5.50–15.29)	< 0.001*
SII^a^	786.94 (449.40–1337.91)	2056.18 (1138.21–3366.66)	< 0.001*
SIRI^a^	2.16 (1.16–3.91)	7.41 (3.89–15.48)	< 0.001*

Abbreviations: AA, acute appendicitis; NLR, neutrophil to lymphocyte ratio; SII, systemic immune‐inflammation index; SIRI, systemic inflammation response index; WBC, white blood cell.

^a^ median (IQR).

^b^ *10^3^/µL.

* indicates statistically significant.

### 3.4 Comparative Analysis of Inflammatory Biomarkers Between Simple and Complicated Acute Appendicitis Participants

There was a statistically significant median SII difference between the SAA and CAA groups. Additionally, the median SIRI of CAA group was significantly higher compared to that of SAA group. The WBC and NLR were also significantly different between the two groups (Table 4).

**Table 4:** CBC parameters analysis of histopathological AA‐positive participants.

Parameters	Mean ± SD/Median (IQR)		*p* value
	SAA (*N* = 34)	CAA (*N* = 39)	
WBC^b^	10.95 ± 4.15^c^	14.66 ± 5.22^c^	0.001*
NLR^a^	6.42 (4.40–9.81)	13.08 (8.73–17.48)	< 0.001*
SII^a^	1188.60 (940.35–2032.74)	3100.59 (2068.66–4282.88)	< 0.001*
SIRI^a^	4.93 (1.82–8.66)	9.71 (5.22–18.67)	< 0.001*

Abbreviations: CAA, complicated acute appendicitis; NLR, neutrophil to lymphocyte ratio; SAA, simple acute appendicitis; SII, systemic immune‐inflammation index; SIRI, systemic inflammation response index; WBC, white blood cell.

^a^ Median (IQR).

^b^ Mean ± SD.

^c^ *10^3^/µL.

* indicates statistically significant.

### 3.5 Diagnostic Accuracy of Inflammatory Biomarkers for Acute Appendicitis

**Figure 2:**
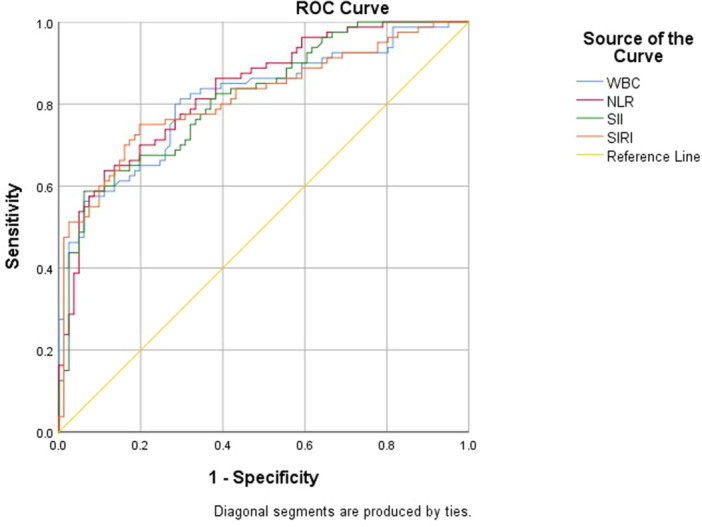
ROC curve analysis of WBC, NLR, SII, and SIRI for the diagnosis of AA.

In this study, SII demonstrated an AUC of 0.816 (95% CI: 0.773–0.895) for diagnosing AA at a cut‐off value of 1929.44 or above. On the other hand, SIRI had an AUC of 0.814 (95% CI: 0.748–0.880) to diagnose AA Similarly, WBC and NLR can diagnose AA with an AUC of 0.814 and 0.834, respectively (Figure 2 and Table 5).

**Table 5:** Diagnostic accuracy of inflammatory biomarkers for AA.

Parameter	Youden index	Cut off value	AUC	95% CI	*p* value	SE (%, 95% CI)	SP (%, 95% CI)	PPV (%, 95% CI)	NPV (%, 95% CI)
WBC	0.516	≥ 8.56^a^	0.814	0.748–0.880	< 0.001	81.3 71.0–89.1	70.4 59.2–80.0	73.0 65.6–79.4	79.2 70.2–86.0
NLR	0.526	≥ 7.97	0.834	0.773–0.895	< 0.001	63.8 52.2–74.2	88.9 80.0–94.8	85.0 75.0–91.5	71.3 64.8–77.0
SII	0.526	≥ 1929.44	0.816	0.773–0.895	< 0.001	58.8 47.2–69.7	93.8 86.2–98.0	90.0 79.8–95.7	68.5 63.8–75.1
SIRI	0.552	≥ 4.20	0.814	0.748–0.880	< 0.001	75.0 64.1–84.0	80.2 69.9–88.3	78.9 70.4–85.6	76.5 68.7–82.8

Abbreviations: AUC, area under the curve; NLR, neutrophil to lymphocyte ratio; NPV, negative predictive value; PPV, positive predictive value; SE, sensitivity; SII, systemic immune‐inflammation index; SIRI, systemic inflammation response index; SP, specificity; WBC, white blood cells.

^a^ *10^3^/µL.

### 3.6 Diagnostic Accuracy of Inflammatory Biomarkers for Complicated Acute Appendicitis

Based on the histopathology results, the 73 participants with confirmed AA were grouped as SAA and CAA to observe the value of CBC parameters for AA severity. Of the 73 confirmed AA, 39 (53.42%) had CAA, and the remaining 34 (46.58%) had SAA.

**Figure 3:**
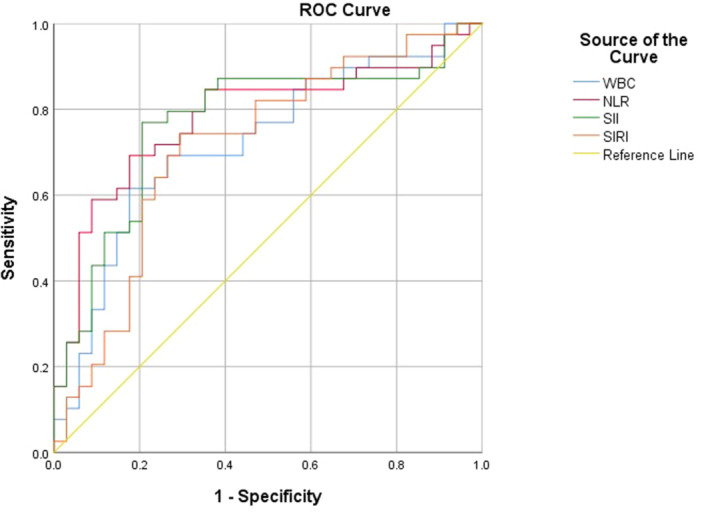
ROC curve analysis of WBC, NLR, SII, and SIRI for the diagnosis of CAA.

In this study, SII and SIRI can predict CAA with an AUC of 0.770 (95% CI: 0.657–0.883) and 0.715 (95% CI: 0.594–0.836). WBC had an AUC of 0.722 (95% CI: 0.604–0.841) to predict CAA. On the other hand, NLR had a predictive value for CAA with an AUC of 0.781 (95% CI: 0.671–0.890) (Figure 3 and Table 6).

**Table 6:** Diagnostic accuracy of inflammatory biomarkers for CAA.

Parameter	Youden index	Cut off value	AUC (95% CI)	*p* value	SE (%, 95% CI)	SP (%, 95% CI)	PPV (%, 95% CI)	NPV (%, 95% CI)
WBC	0.439	≥ 13.87^a^	0.722 (0.604–0.841)	0.001	61.5 (44.6–76.6)	82.4 65.5–93.2	80.0 65.0–89.6	65.1 54.9–74.1
NLR	0.516	≥ 10.84	0.781 (0.671–0.890)	< 0.001	69.2 52.4–83.0	82.4 65.5–93.2	81.8 67.9–90.6	70.0 58.7–79.3
SII	0.563	≥ 2056.18	0.770 (0.657–0.883)	< 0.001	76.9 60.7–88.9	79.4 62.1–91.3	81.8 68.4–89.5	75.0 62.3–84.5
SIRI	0.449	≥ 6.13	0.715 (0.594–0.836)	0.002	74.4 57.9–87.0	67.6 49.5–82.6	72.5 61.1–81.6	69.7 56.2–80.5

Abbreviations: AUC, area under the curve; NLR, neutrophil to lymphocyte ratio; NPV, negative predictive value; PPV, positive predictive value; SE, sensitivity; SII, systemic immune‐inflammation index; SIRI, systemic inflammation response index; SP, specificity; WBC, white blood cells.

^a^ *10^3^/µL.

## 4 Discussion

The need for cost‐effective diagnostic adjuncts in AA has led to the investigation of several inflammatory markers. While markers like DNI, C‐reactive protein/albumin ratio (CAR), procalcitonin (PCT), hemoglobin, albumin, lymphocyte, platelet score (HALPs) [46], and hepcidin [47] showed promise as independent predictors of AA, their laboratory measurement is often more expensive or less available in resource‐constrained settings. In contrast, CBC‐derived indices like NLR, SII, and SIRI offer accessible and effective diagnostic alternatives.

Our study demonstrated that WBC count remains a reliable indicator for AA, supported by the physiological immune response to appendiceal obstruction [48]. The current study reported an acceptable diagnostic power of WBCs for AA, which is supported by a study done in Pakistan [49], but excellent values were reported in some studies using healthy controls [50]. We attribute this variation to our “sick controls” (patients with non‐AA abdominal pain), as they mimic the inflammatory profile of AA; the biological distinction between the groups is narrowed. Furthermore, the significant leukocytosis observed in our CAA group reflects the transition from localized inflammation to systemic infection, likely triggered by increased bacterial load and cytokine release [51, 52]. The WBC count showed moderate potential in differentiating CAA from SAA.

Moreover, the NLR was significantly associated with both AA and its severity. An elevated NLR reflects the dual‐faceted nature of the systemic immune response, neutrophilia (innate immune activation) and lymphocytopenia (a shift or exhaustion in adaptive immunity) [53, 54]. The NLR had good diagnostic value for AA, and it predicts CAA only fairly. Its modest sensitivity of NLR (63.8%) to diagnose AA highlights that this parameter should not be used as a standalone “rule‐out” tool, instead with other inflammatory biomarkers and clinical findings.

Similarly, both SII and SIRI demonstrated significant median difference across all study groups, reflecting a pro‐inflammatory state [55]. SII integrates neutrophils and platelets (pro‐inflammatory) with lymphocytes (regulatory), capturing the role of platelets in leukocyte migration and cytokine release [20, 56]. On the other hand, SIRI integrated clinically available peripheral neutrophil, monocyte, and lymphocyte counts. Monocytes contribute to the inflammatory cascade by releasing cytokines like IL‐1 and IL‐6 [56]. The higher SII and SIRI in CAA relative to SAA in this study are in keeping with other studies by Kiss et al. and Yarkaç et al. [57, 58]. Both SII and SIRI had moderate diagnostic performance for AA, but differentiated SAA from CAA only fairly. While some studies, such as Ertekin and Acar [19], reported a high potential value of SII and SIRI to predict CAA, our “moderate” accuracy may be due to a higher prevalence of advanced disease in our referral‐based cohort, which narrows the discriminatory window. A higher prevalence of CAA, often seen in referral centers where patients present with advanced disease, leads to a biological overlap between high‐grade SAA and early‐stage complications, narrowing the discriminatory window for inflammatory biomarkers. Conversely, in populations where SAA is the predominant finding, the diagnostic gap between simple and complicated states is often wider, resulting in higher AUC values.

In this study, compared to the present study, a lower diagnostic value of SII and SIRI for AA and predicting CAA was reported by Siki et al. [28]. SII demonstrated poor predictive value according to the findings of a study by Çoşkun et al. [59]. These inconsistencies may be primarily attributed to fundamental differences in immune physiology between pediatric and adult populations, as studies by Siki et al. and Çoşkun et al. include only pediatrics. Pediatric immune systems are characterized by high reactivity; non‐surgical conditions such as viral mesenteric adenitis can trigger significant elevations in neutrophils and monocytes, creating substantial “biological noise” that reduces the specificity of composite inflammatory indices. The rapid pathological progression of AA in children may outpace the systemic hematological response, leading to a diagnostic lag.

The diagnostic accuracy of NLR, SII, and SIRI observed in our study aligns with the growing body of literature on composite inflammatory scores. For instance, the BIDIAP, a novel diagnostic index developed by Arredondo Montero et al. [60], has been recognized as an excellent diagnostic tool for pediatric AA (AUC = 0.9734). It integrates SII, appendicular caliber, and presence of peritoneal irritation [26, 60]. By integrating multiple cellular lineages (neutrophils, lymphocytes, and platelets), these indices capture the complex systemic inflammatory response similarly to established scores but offer a more cost‐effective and rapid alternative for early surgical triage.

Beyond AA, SII, and SIRI have demonstrated significant value in the diagnostic and prognostic assessment of various malignancies. In patients with metastatic pancreatic cancer receiving chemotherapy, Akar et al. [61] identified SIRI as an independent prognostic biomarker significantly associated with survival outcomes. Similarly, Troise et al. [31] suggested that these indices could serve as routine preoperative supportive tools for detecting occult neck lymph node metastases in early‐stage oral cavity carcinomas. Furthermore, Abbate et al. [32] reported that SIRI provides valuable diagnostic support alongside Fine‐Needle Aspiration Cytology (FNAC), particularly in resolving equivocal cases of salivary gland tumors. Recently, Ilkkilic and Sen [62] identified SIRI as an independent prognostic factor of Hodgkin Lymphoma. As the investigators suggested, increased SIRI is associated with shorter progression‐free and overall survival. Collectively, these findings underscore the versatility of these markers in identifying systemic inflammatory signatures across diverse pathological states.

The clinical value of SII and SIRI would be greatest in resource‐limited settings, where access to advanced imaging or formal scoring systems is restricted. The Alvarado score and other clinical scores remain a cornerstone of clinical diagnosis for AA; however, their reliance on subjective physical signs can lead to diagnostic uncertainty, especially in distinguishing between SAA and CAA [63]. The clinical significance of our findings is particularly relevant for healthcare systems in low‐ and middle‐income countries (LMICs), such as Ethiopia. In many of these settings, imaging modalities are often unavailable, costly, or geographically inaccessible to rural populations.

### 4.1 Strength and Limitations of the Study

Being the first study in Ethiopia, which may serve as baseline information for further studies, was one of the strengths of the current study. Furthermore, the study uses histopathology examination for severity determination, which allows us to categorize the participant as NA, SAA, or CAA. However, several limitations must be acknowledged. A primary limitation of this study was that formal age stratification was not statistically feasible due to the limited number of pediatric participants under 18 years of age (*n* = 20 for AA and *n* = 12 for non‐AA). Future multi‐center studies with larger, age‐balanced cohorts are essential to establish stratified “optimal cutoffs”. The subgroup comparison of SAA and CAA also involved a relatively small number of participants (34 and 39, respectively). Consequently, our findings regarding the differentiation of SAA and CAA should be interpreted with caution and validated in larger, multi‐center prospective studies. Another potential limitation may be related to the diagnostic classification of the non‐AA group. The non‐AA group was primarily defined by US findings and clinical evaluation. While clinical follow‐up until discharge was used to rule out AA in the no‐AA group, the absence of routine CT may represent a limitation in definitively ruling out every occult case of appendiceal inflammation. While the use of consecutive sampling minimized selection bias within our study period, this approach is susceptible to temporal bias. Our results may not account for seasonal variations in the incidence of gastrointestinal conditions. Seasonal fluctuations in endemic diseases such as waterborne gastroenteritis during the late rainy months could elevate baseline systemic inflammatory markers in the symptomatic control group, and this may influence baseline inflammatory markers. Thus, future studies utilizing probability‐based sampling technique are needed to validate the generalizability of these findings.

## 5 Conclusion

In conclusion, both SII and SIRI are cost‐effective, easy to calculate, and can be determined at the bedside of the patients based on CBC results. The SII and SIRI offer a multi‐dimensional view of the systemic response. While imaging remains the gold standard, its availability in distant or low‐resource locations is often limited. In such contexts, SII and SIRI may not replace imaging but serve as adjunctive tools to assist in clinical decision‐making and risk stratification. Although their independent diagnostic accuracy for identifying complicated cases is moderate, these cost‐effective parameters could assist clinicians in identifying high‐risk patients who may require prioritized surgical consultation alongside standard clinical assessment. The clinical value of NLR, SII, and SIRI in the diagnosis of AA and determining its severity is enhanced by combining them with other conventional inflammatory indices such as CRP. Moreover, the clinical utility of these indices warrants further validation through larger multi‐center prospective studies focused on establishing standardized diagnostic cutoff values.

## Author Contributions

**Sintayehu Admas:** conceptualization, investigation, writing – original draft, methodology, validation, visualization, writing – review and editing, software, formal analysis, data curation. **Bisrat Birke Teketelew:** conceptualization, methodology, validation, visualization, writing – review and editing, software, formal analysis, data curation, supervision. **Nigusie Alemu:** software, visualization, writing – review and editing, validation, formal analysis, data curation, methodology. **Berhanu Woldu:** methodology, data curation, writing – review and editing, validation, visualization. **Lyusira Marelgn:** methodology, validation, visualization, writing – review and editing, formal analysis, data curation, software. **Getu Girmay:** methodology, validation, visualization, writing – review and editing, software, formal analysis, data curation. **Arega Zenaw:** methodology, visualization, validation, writing – review and editing, software, formal analysis, data curation. **Abebe Birhanu:** methodology, validation, visualization, writing – review and editing, software, formal analysis, data curation. **Yemataw Gelaw:** methodology, validation, visualization, writing – review and editing, software, formal analysis, data curation, supervision, conceptualization.

## Funding

The authors have nothing to report.

## Ethics Statement

This study was reviewed and approved by the Ethics Committee of the School of Biomedical and Laboratory Sciences, University of Gondar, Gondar, Ethiopia (Ref. No: SBMLS/812/2024). The study was conducted as per the Helsinki Declaration for Biomedical Research [38].

## Consent

All adult study participants and legal guardians provided informed written consent prior to study enrollment.

## Conflicts of Interest

The authors declare no conflicts of interest.

## Transparency Statement

The lead author, Sintayehu Admas, affirms that this manuscript is an honest, accurate, and transparent account of the study being reported; that no important aspects of the study have been omitted; and that any discrepancies from the study as planned (and, if relevant, registered) have been explained.

## Supporting information


**Table S1:** Variable assessment questionnaire.

**Supporting File:** Checklist: STROBE statement for observational studies.

## Data Availability

The corresponding author will make the data sets that were used throughout this study available to the interested party upon reasonable request.
