# Supplementary material for: Diagnostic Accuracy of Inflammatory Biomarkers in Differentiating Acute Appendicitis From Other Acute Abdomen and Predicting Disease Severity: A Prospective Comparative Cross‐Sectional Study
Source: Health Sci Rep. 2026 May 25;9(6):e72562. doi: 10.1002/hsr2.72562 (PMC13240539; doi:10.1002/hsr2.72562)
Supplement: Supplementary file 1 — Table S1: Variable assessment questionnaire. [file HSR2-9-e72562-s002.docx]

**S1 Table**: Study variable assessment questionnaire

| **Socio-demographic characteristics** | | |
| --- | --- | --- |
| No | Questions | Response |
| 1 | Age (in years) | __________________ |
| 2 | Sex | A. Male B. Female |
| **Clinical characteristics** | |  |
| 3 | Migratory pain to RLQ | A. Yes B. No |
| 4 | Anorexia | A. Yes B. No |
| 5 | Nausea and vomiting | A. Yes B. No |
| 6 | RLQ tenderness | A. Yes B. No |
| 7 | Rebound tenderness | A. Yes B. No |
| 8 | Fever, >37.5°C | A. Yes B. No |
| 9 | Leukocytosis, WBC>10*10^9^/µL | A. Yes B. No |
| **CBC Parameters** | | Result |
| 1 | WBC (*10^3^/µL) |  |
| 2 | ANC (*10^3^/µL) |  |
| 3 | ALC (*10^3^/µL) |  |
| 4 | AMC (*10^3^/µL) |  |
| 5 | Platelet count |  |
| 6 | NLR |  |
| 7 | SII |  |
| 8 | SIRI |  |

RLQ; Right Lower Quadrant, WBC: White Blood Cell, ANC: Absolute Neutrophil Count, ALC: Absolute Lymphocyte Count, AMC: Absolute Monocyte Count, NLR: Neutrophil to Lymphocyte Ratio, SII: systemic immune-inflammation index, SIRI: systemic inflammation response index
