# Supplementary material for: Diagnostic Accuracy of Inflammatory Biomarkers in Differentiating Acute Appendicitis From Other Acute Abdomen and Predicting Disease Severity: A Prospective Comparative Cross‐Sectional Study
Source: Health Sci Rep. 2026 May 25;9(6):e72562. doi: 10.1002/hsr2.72562 (PMC13240539; doi:10.1002/hsr2.72562)
Supplement: Supplementary file 2 — Supporting File: Checklist: STROBE statement for observational studies. [file HSR2-9-e72562-s001.docx]

S1 Checklist. STROBE checklist for observational study

|  | Item No. | Recommendation | Page No | Relevant text from manuscript |
| --- | --- | --- | --- | --- |
| **Title and abstract** | 1 | (a) Indicate the study’s design with a commonly used term in the title or the abstract | 1 | Cross-sectional study |
|  |  | (b) Provide in the abstract an informative and balanced summary of what was done and what was found | 1-2 |  |
| Introduction | | | |  |
| Background/rationale | 2 | Explain the scientific background and rationale for the investigation being reported | 3-6 |  |
| Objectives | 3 | State specific objectives, including any pre-specified hypotheses | 6 |  |
| Methods | | | |  |
| Study design | 4 | Present key elements of study design early in the paper | 6 |  |
| Setting | 5 | Describe the setting, locations, and relevant dates, including periods of recruitment, exposure, follow-up, and data collection | 6 and 9-10 |  |
| Participants | 6 | Cross-sectional study—Give the eligibility criteria, and the sources and methods of selection of participants | 7-8 |  |
| Variables | 7 | Clearly define all outcomes, exposures, predictors, potential confounders, and effect modifiers. Give diagnostic criteria, if applicable |  | NA |
| Data sources/ measurement | 8* | For each variable of interest, give sources of data and details of methods of assessment (measurement). Describe comparability of assessment methods if there is more than one group | 10-11 | Venous blood was collected by professional laboratory technologist using EDTA. Mindray BC-5150 (*Shenzhen, P.R. China*) was used for CBC analysis. Tissue sections are stained with H/E stain and examined microscopically by pathologists. |
| Bias | 9 | Describe any efforts to address potential sources of bias |  | NA |
| Study size | 10 | Explain how the study size was arrived at | 9 | The sample size was determined using the formula for estimating accuracy index (AUC) by taking the pre-determined AUC from the previous study, considering the 95% CI, and 5% margin of error (d). |

| Quantitative variables | 11 | Explain how quantitative variables were handled in the analyses. If applicable, describe which groupings were chosen and why | 12 |  |
| --- | --- | --- | --- | --- |
| Statistical methods | 12 | (a) Describe all statistical methods, including those used to control for confounding | 12 |  |
|  |  | (b) Describe any methods used to examine subgroups and interactions |  | NA |
|  |  | (c) Explain how missing data were addressed |  | NA |
|  |  | (d) Cross-sectional study—If applicable, describe analytical methods taking account of sampling strategy |  |  |
|  |  | (e) Describe any sensitivity analyses |  | NA |
| Results | | | | |
| Participants | 13* | (a) Report numbers of individuals at each stage of study—eg numbers potentially eligible, examined for eligibility, confirmed eligible, included in the study, completing follow-up, and analysed | 12 | A total of 161 study participants were enrolled in this study. There were 81 non-AA and 80 AA participants. |
|  |  | (b) Give reasons for non-participation at each stage |  | NA |
|  |  | (c) Consider use of a flow diagram |  | NA |
| Descriptive data | 14* | (a) Give characteristics of study participants (eg demographic, clinical, social) and information on exposures and potential confounders | 12-15 |  |
|  |  | (b) Indicate number of participants with missing data for each variable of interest |  | NA |
| Outcome data | 15* | Cross-sectional study—Report numbers of outcome events or summary measures | 16-21 |  |
| Main results | 16 | (a) Give unadjusted estimates and, if applicable, confounder-adjusted estimates and their precision (eg, 95% confidence interval). Make clear which confounders were adjusted for and why they were included |  | NA |
|  |  | (b) Report category boundaries when continuous variables were categorized |  | NA |
|  |  | (c) If relevant, consider translating estimates of relative risk into absolute risk for a meaningful time period |  | NA |

Continued on next page

| Other analyses | 17 | Report other analyses done—eg analyses of subgroups and interactions, and sensitivity analyses |  | NA |
| --- | --- | --- | --- | --- |
| Discussion | | | | |
| Key results | 18 | Summarise key results with reference to study objectives | 21-24 | Inflammatory biomarkers such as WBC, NLR, SII and SIRI could serve as an adjunct diagnostic tool to differentiate AA from non-AA abdominal pain especially in resource-limited areas where imaging modalities are not accessible. |
| Limitations | 19 | Discuss limitations of the study, taking into account sources of potential bias or imprecision. Discuss both direction and magnitude of any potential bias | 24-25 | Formal age stratification was not statistically feasible due to the limited number of pediatric participants under 18 years of age. The non-AA group was primarily defined by US findings and clinical evaluation. |
| Interpretation | 20 | Give a cautious overall interpretation of results considering objectives, limitations, multiplicity of analyses, results from similar studies, and other relevant evidence | 21-25 |  |
| Generalisability | 21 | Discuss the generalisability (external validity) of the study results | 25-26 |  |
| Other information | |  | | |
| Funding | 22 | Give the source of funding and the role of the funders for the present study and, if applicable, for the original study on which the present article is based |  | NA |
| na |  |  |  |  |
